# Localization of Pyelonephritis by ^68^Ga-FAPI PET CT

**DOI:** 10.22038/AOJNMB.2022.64168.1450

**Published:** 2023

**Authors:** Rajlaxmi Rangrao Jagtap, Shailendra Vasant Savale, Gauri Shridhar Khajindar, Shrikant Vasantrao Solav

**Affiliations:** Spect Lab, Pune, India

**Keywords:** ^68^Ga-FAPI, Pyelonephritis, ^ 99m^Tc-DMSA

## Abstract

Fibroblast activation protein inhibitor (FAPI) is a quinoline-based membrane-bound glycoprotein enzyme that is not usually expressed in normal adult tissues, except for the myometrium and sometimes the pancreas. Its expression increases in inflammation and cancer-associated fibroblasts (CAF). As FAPI is a new molecule with a promising future, presented here is a case report of uncontrolled diabetes with abdominal pain that showed features of inflammation in the kidneys. The patient had been previously diagnosed with maxillary sinusitis with Aspergillus niger and was receiving antibiotic treatment. The urine culture performed later was negative, and the patient was referred for ^18^F-fluorodeoxyglucose (^18^F-FDG) PET Computed Tomography (CT) to look for the focus of infection. However, as blood sugar was 500 mg/dL, ^18^F-FDG PET CT could not be performed. Therefore, ^68^Ga-FAPI PET CT was run instead after taking the patient’s written informed consent. The ^99m^Tc-dimercaptosuccinic acid scan performed on another day confirmed the presence of pyelonephritis bilaterally.

In situations where FDG cannot be used because of hyperglycemia, ^68^Ga-FAPI PET CT scan may be considered an alternative in the detection of occult infection or inflammation, as demonstrated in this case report.

## Introduction

 The ^68^Ga Fibroblast activation protein inhibitor (^68^Ga-FAPI) tracer localizes in various neoplasms due to its cancer-associated fibroblast activity (CAF) (1). Such fibroblasts are associated with inflammatory and infectious conditions, such as tuberculosis (2) and cholecystitis (3). Immunoglobulin G4 (IgG4)-related disease is characterized by the infiltration of various tissues with IgG4-rich lymphoplasmacytic fibrotic tissue, which has been reported to express CAF (4).

 The time-tested molecule for ^18^F-fluoro-deoxyglucose (^18^F-FDG) cancer imaging requires four to six hours of fasting state. In addition to tumor imaging, ^18^F-FDG PET Computed Tomography (CT) scan can be used for the evaluation of pyrexia of unknown origin caused by occult infections, as well as inflammatory processes; however, uncontrolled diabetes limits its usage. A variety of infections, including but not limited to pyelonephritis, tuberculosis, osteomyelitis, and inflammatory processes, such as sarcoidosis, can be detected through ^18^F-FDG PET CT scan. The FDG also accumulates in the kidneys, thereby limiting its usage in kidney disorders.

 The ^68^Ga-FAPI PET CT, on the other hand, does not require fasting. The patient discussed here was referred for ^18^F-FDG PET CT in view of recurrent unexplained abdominal pain, dysuria, and weight loss. As the blood sugar level was more than the recommended value of 200mg/dL, a decision was made to perform ^68^Ga-FAPI PET CT, which provided traces of pathological involvement of the kidneys.

***Case History***

 The patient was a 57-year-old woman with long-standing diabetes and a history of surgery for right maxillary sinusitis. She had undergone inferior turbinoplasty in May 2021. Histology showed chronic sinusitis, and the culture revealed the growth of Aspergillus niger along with chronic sinusitis. She presented in August 2021 with pain in the abdomen, recent weight loss of 3 kg, and an increased frequency of micturition. Ultrasonography of the abdomen and pelvis did not show any significant abnormality. However, empirical antibiotics were prescribed for the patient. The initial urine microscopy showed 1-2 pus cells/high power field. The ^18^F-FDG PET CT was advised in view of unexplained abdominal pain and weight loss. 

 As her blood sugar was in the range of 500 mg/dl, ^18^F-FDG PET CT could not be performed. In addition, since the patient was from a distant rural area, she couldn’t revisit after controlling the blood sugar; therefore, she was offered ^68^Ga-FAPI PET CT scan. Imaging to assess glomerular filtration rate (GFR) could not be performed either as serum creatinine and urea were normal.

 The test was performed using 3.6 millicuries of ^68^Ga-FAPI, and the imaging was conducted using Siemens Biograph (Siemens Healthineers, Germany) PET CT system 60 minutes after injection. There was an increased inhomogeneous distribution of the tracer in both kidneys. Magnetic resonance imaging (MRI) of kidneys showed multiple cortical-based wedge-shaped areas of restricted diffusion in both kidneys. Urine examination showed 50-60 pus cells/high power field and a negative urine culture. The ^99m^Tc-dimercaptosuccinic acid (^99m^Tc-DMSA) scan was performed the next day after injection of 2.5 millicuries. The imaging was performed on Siemens EVO excel gamma camera (Siemens Healthineers, Germany) using low energy, high resolution, and pinhole collimator at 3 h post tracer administration. The scan showed a normal tracer uptake by both kidneys without any loss of volume. There were multiple cortical-based cold areas. Compared to the ^68^Ga-FAPI PET CT and MRI findings, a working diagnosis of pyelonephritis was made.

## Discussion

 Pyelonephritis is the inflammation of the kidneys as a result of an upper urinary tract infection. It may be unilateral or bilateral and can spread from the lower urinary tract to the kidneys as an ascending infection or less commonly, through the hematogenous route. Pyelonephritis can be complicated by factors, such as uncontrolled diabetes, pregnancy, immunocompromised status, anatomical abnormalities, or renal failure. The absence of the aforementioned factors indicates that pyelonephritis is uncomplicated (5). The commonest symptoms of pyelonephritis are fever with chills, nausea or vomiting, and flank pain. These may be associated with dysuria, pyuria, and increased frequency, as well as the urgency of urination. Gram-negative bacteria, such as Escherichia coli, are the most common organisms to cause pyelonephritis. It can also be caused by Vesico-ureteric reflux or obstructive uropathy, such as ureteric calculi (5). Urine analysis shows the presence of pyuria. Complete blood counts show elevated white blood cell counts (5, 6). Imaging can be performed by ultrasonography, CT), ^99m^Tc-DMSA scan, or MRI. The ^18^F-FDG PET CT may also be considered for evaluating the focus of infection.

 The ^99m^Tc-DMSA scan is an investigation used for functional and morphological assessment of the kidneys. It is very sensitive for the detection of renal scarring and assessment of differential function. In pyelonephritis, the scan findings show cortical defects without any loss of volume. The scan may be repeated to assess the progression of cortical defects (7).

 In MRI, the affected areas are hypointense on T1-weighted images and hyperintense on T2 weighted images with restricted diffusion (6). 

The ^18^F-FDG PET CT has some utility in the imaging of pyelonephritis. Involved areas show increased cortical FDG uptake (8). 

 As for the patient under discussion, ^18^F-FDG PET CT could not be performed as the blood sugar was high, and it was logistically difficult for the patient to return from a distant rural area after controlling the blood sugar. Therefore, ^68^Ga-FAPI PET CT scan was performed after taking the patient’s informed written consent.

 The ^68^Ga-FAPI detects the expression of fibroblast activity (1). Increased fibroblast activity is seen in malignant tumors, as well as non-neoplastic states, such as infection and inflammation (1), making FAPI scan useful for diagnosis. Studies have described the increased uptake of ^68^Ga-FAPI extrapulmonary tuberculosis (2), as well as the inflammatory etiology, such as cholecystitis (3). 

**Figure 1:**
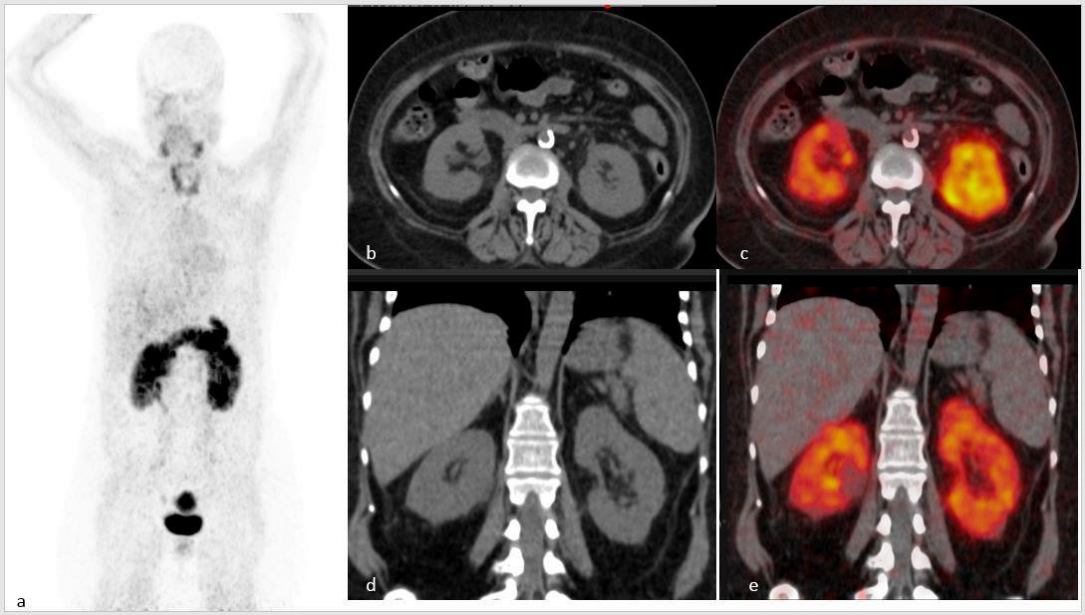
MIP image of ^68^Ga-FAPI PET CT shows increased radiotracer uptake in both kidneys. Physiological tracer uptake in the pancreas and myometrium. Increased tracer uptake in the thyroid is likely to be related to thyroiditis (**a**). Trans-axial CT and fused PET CT images (**b** and** c**, respectively), as well as coronal CT and fusion PET CT images. (**d** and **e**) show avid inhomogeneous tracer uptake in both kidneys suggestive of inflammatory etiology

 The ^68^Ga-FAPI PET CT of the patient showed an increased tracer uptake in both kidneys without any loss of renal volume. The incidentally-observed increased uptake in the thyroid gland was likely to be related to thyroiditis (9). Physiological tracer uptake was noted in the pancreas and myometrium (Figure 1).

**Figure 2:**
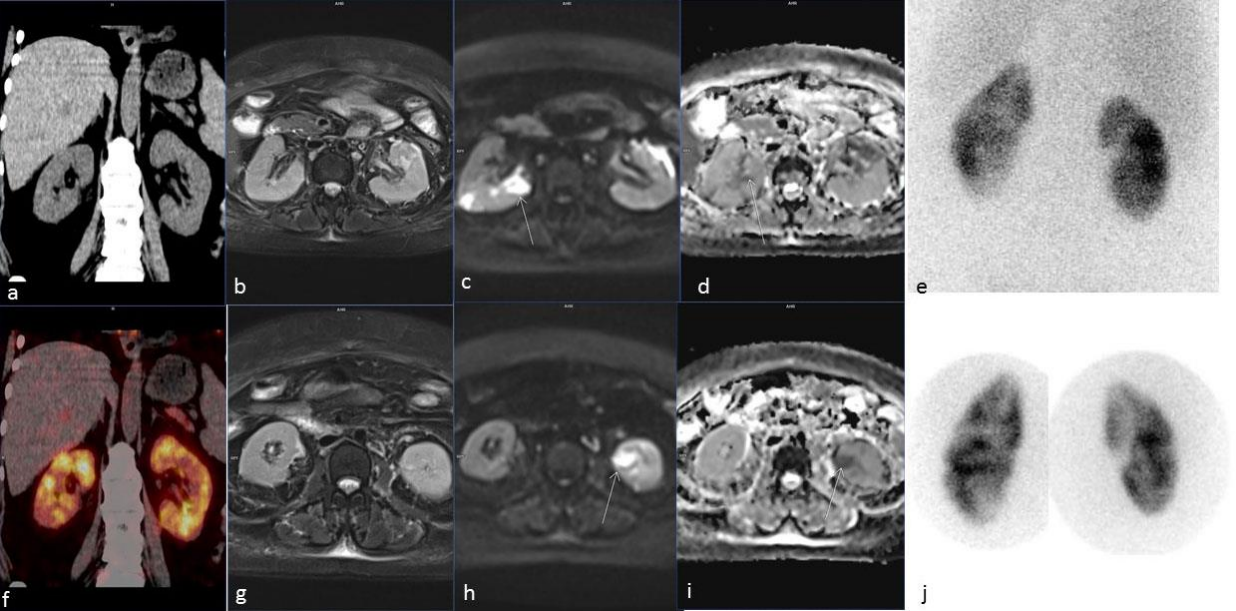
Coronal CT (**a**) and fusion ^68^Ga-FAPI PET CT (**f**) images show diffuse increased tracer uptake in both kidneys. The T2-Weighted sequence of MRI (**b** and **g**) shows increased cortical-based signal intensities. Diffusion-weighted images show hyperintense signals in high B **(c** and **h**) appearing dark on apparent diffusion coefficient maps (**d** and **i**). Dimercaptosuccinic acid planar images with LEHR (**e**) and pinhole (**j**) collimators show the normal size of both kidneys with cortical irregularities and multiple cortical-based areas of photon deficiencies compatible with pyelonephritis

 The MRI showed cortical wedge-shaped areas of restricted diffusion in bilateral kidneys (Figures 2a- d, and f- i). The ^99m^Tc-DMSA scan shows corresponding wedge-shaped cortical defects (Figures 2e and j).

 A working diagnosis of pyelonephritis (diabetic renal papillary necrosis) was established based on the above findings. A differential diagnosis of renal fibrosis was also considered (10).

 From the above-discussed case, it can be inferred that ^68^Ga-FAPI PET CT may be considered for the detection of infective or inflammatory foci (pyelonephritis/fibrosis in the above-described case), especially in uncontrolled diabetes when ^18^F-FDG PET CT may not be possible.

## Declaration of the Patient’s Consent

 The authors certify that they have obtained all appropriate patient consent forms. In the form, the patient has given her consent for her images and other clinical information to be reported in the journal. The patient understands that her name and initials will not be published and due efforts will be made to conceal her identity, but anonymity cannot be guaranteed.

## Conflict of Interest

 There are no conflicts of interest to declare.
